# A Field-Portable Cell Analyzer without a Microscope and Reagents

**DOI:** 10.3390/s18010085

**Published:** 2017-12-29

**Authors:** Dongmin Seo, Sangwoo Oh, Moonjin Lee, Yongha Hwang, Sungkyu Seo

**Affiliations:** 1 Department of Electronics and Information Engineering, Korea University, Sejong 30019, Korea; ehdals20907@korea.ac.kr (D.S.); swoh@kriso.re.kr (S.O.); 2 Maritime Safety Research Division, Korea Research Institute of Ships & Ocean Engineering, Daejeon 34103, Korea; moonjin.lee@kriso.re.kr; 3 Department of Electro-Mechanical Systems Engineering, Korea University, Sejong 30019, Korea

**Keywords:** lens-free cell analyzer, automatic cell analysis algorithm, reagent-free, cell counting, viability analysis, Lens-Free Shadow Imaging Technology (LSIT)

## Abstract

This paper demonstrates a commercial-level field-portable lens-free cell analyzer called the NaviCell (No-stain and Automated Versatile Innovative cell analyzer) capable of automatically analyzing cell count and viability without employing an optical microscope and reagents. Based on the lens-free shadow imaging technique, the NaviCell (162 × 135 × 138 mm^3^ and 1.02 kg) has the advantage of providing analysis results with improved standard deviation between measurement results, owing to its large field of view. Importantly, the cell counting and viability testing can be analyzed without the use of any reagent, thereby simplifying the measurement procedure and reducing potential errors during sample preparation. In this study, the performance of the NaviCell for cell counting and viability testing was demonstrated using 13 and six cell lines, respectively. Based on the results of the hemocytometer (*de facto* standard), the error rate (ER) and coefficient of variation (CV) of the NaviCell are approximately 3.27 and 2.16 times better than the commercial cell counter, respectively. The cell viability testing of the NaviCell also showed an ER and CV performance improvement of 5.09 and 1.8 times, respectively, demonstrating sufficient potential in the field of cell analysis.

## 1. Introduction

Cell counting and viability testing are indispensably integrated in the field of modern biotechnology and biomedical research [1,2,3]. Both fundamental analyses can confirm the sample concentration in a cell culture process and identify a cell proliferation cycle, allowing researchers to schedule cell-based experiments and to check for contamination of an incubator or cells [4,5]. Hemocytometer analysis is a method in which a microscope is used to visually identify stained cells, followed by manually counting the identified cells, which is the most common method for cell counting and viability testing [6,7,8]. While manual counting using the hemocytometer has the advantage of low cost and versatility, it is intrinsically time-consuming and labor intensive [9,10]. Furthermore, manual counting has the potential for subjective errors by operators during the process of counting the cells. Hemocytometer analysis, however, is still the *de facto* standard for cell counting and viability testing and is used by approximately 70% of analysts [11,12].

The continuous growth of modern biotechnology and biomedical research requires the cell counting and viability testing to quickly and easily analyze large volumes of samples and data [13,14,15]. This has led to the development of automated cell counters that reinforce the disadvantages of traditional manual counting and comprise the mainstream methods currently available/used in the market. The global cell counter market is expected to grow by an average of 9% per annum from 2015 to 2020 and reach USD 8.6 billion by 2020 [16]. Early automated cell counters based on flow cytometry or *Coulter* counter provide accurate analysis of individual cells and are also suitable for large sample handling [17,18,19]. However, these instruments are extremely expensive, bulky and require specialists to operate [20,21]. To meet the need to significantly reduce the cost and volume of automatic cell counting facilities, Invitrogen, BioRAD, Nexcelom and ORFLO have recently released bench-top based automated cell counters, such as Countess, TC10, MINI and Mozi, respectively. Despite their compact size, all commercial counters are still based on the microscopy technique that requires many expensive optical lenses and components [22]. More importantly, to use the commercial instruments, sample pretreatment using color or fluorescence dyes must be carried out for cell counting and viability testing, causing potential errors due to increased pipetting from complicated test procedures [23,24].

**Figure 1:**
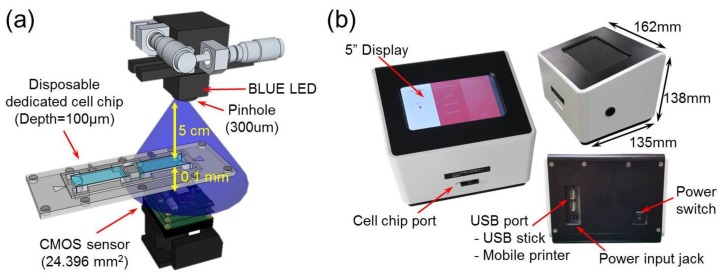
Lens-free cell analyzer, NaviCell. (**a**) The optical part consists of a blue LED, a 300 μm pinhole for coherent illumination to a sample chip and a CMOS image sensor for acquiring images from the sample; (**b**) For convenient usability, a 5-in. touch display is mounted at the top of the device, which allows for the reporting of quantitative results and for viewing images of the samples without additional equipment. The NaviCell is operated by a proprietary program based on an Android system. Samples for analysis are prepared on a disposable chip which was developed to increase the shadow intensity of the cell.

The complete cell analyzer reported in this study is based on the lens-free shadow imaging technique (LSIT), which can analyze the state of cells or particles in real time by imaging the intrinsic shadows, i.e., diffraction patterns [25,26]. In order to analyze cells, techniques for identifying velocity changes between consecutive images [27] and learning the image patterns of individual cells stained by machine learning [28] have been studied recently. In this study, it was demonstrated through various cell lines that cell counting and viability analysis were possible without using reagents, with the peak-to-peak distance parameter extracted by profiling the single cell diffraction pattern. The diffraction patterns can be obtained with a light emitting diode (LED) illuminator, complimentary metal-oxide semiconductor (CMOS) image sensor and pinhole (300 μm); thus, optical lenses and additional complex stages are not required (Figure 1a). The 1/2.5-in. CMOS image sensors were purchased from Micron Technology, Inc. (MT9P031, USD 14 per chip, Boise, ID, USA) and blue LEDs with dominant wavelength of 450–490 nm were purchased from Harvatek Co., Ltd. (USD 3 per chip, Hsinchu City, Taiwan). The NaviCell is designed to fix the LED light source, CMOS image sensor and pinhole using a metal frame called the Chip Bed and the disposable cell chip can be inserted into the middle of the Chip Bed. Since the manufacturing tolerance of the Chip Bed is 0.01 mm, the change in the shadow pattern due to the distance variation between the image sensor and the sample is negligible. Consequently, it is possible to miniaturize the entire system to make it smaller than commercial bench-top instruments and to obtain a wider field of view (FOV), which is unattainable with conventional optical lenses. In addition, since the system recognizes the state of cells or particles individually through the diffraction patterns, cell counting and viability testing can be performed without the use of reagents. The NaviCell is equipped with a reagent-free analysis algorithm that automatically calculates cell count and cell viability according to parameters introduced for the analysis of image contrast. As shown in Figure 1b, the NaviCell has a width, depth, height and weight of 162 mm, 135 mm, 138 mm and 1.02 kg, respectively. It has a 5-in. touch-type liquid crystal display at the top, a cell chip port at the front and a USB interface port at the rear. These configurations are controlled by a custom-built motherboard equipped with an i.MX6 dual processor (Freescale Semiconductor, Inc., Austin, TX, USA). The mainboard is ported to the Android operating system and operated by the developed Android-based analysis program. This analyzer is a complete system, whereby cell samples are automatically analyzed after inserting the cell chip into the cell chip port and selecting the measurement options on the display. It is the smallest, lightest and relatively fastest analysis cell counter ever developed. It also has no need for reagents and offers the widest FOV for accurate analysis. Furthermore, the motherboard and hardware configurations have operating voltages less than 5 V, allowing the adapter to be replaced by a battery for portability. Specifications of the NaviCell are listed in the Table 1 along with those of commercial instruments.

The performance of the NaviCell was verified by comparisons with a hemocytometer and a commercial cell counter. First, 13 cell lines (A549, BT474, CHO, COS7, HELA, HL60, HT29, L929, MDA-MB-231, PC3, SK-BR-3, SWRC-GRO and U87) were analyzed in terms of the coefficient of variation (CV, %) and the error rate (ER, %) of the measurement results based on the data acquired with the hemocytometer. Next, the measurement results of the mammalian cells of six cell lines (BT474, L929, MDA-MB-231, THP-1, SWRC-GRO and U87) were compared in order to analyze the cell viability. Note that every cell line was tested without any reagents needed for the NaviCell, whereas specific dyeing procedures were required for both the hemocytometer and the commercial cell counter.

## 2. Materials and Methods

### 2.1. Materials

For standard cell counting, an optical microscope (S39) was used, which was purchased from MICro Scopes Inc. (St. Louis, MO, USA). A PAXcam 2+ (Villa Park, IL, USA) camera and the commercial cell counter (Countess II) were purchased from Thermo Fisher Scientific (Waltham, MA, USA). Two types of disposable cell chips for the hemocytometer and the commercial cell counter were purchased from NanoEntek (Seoul, Korea). The reagents, including Dulbecco Modified Eagle Medium (DMEM), Roswell Park Memorial Institute (RPMI) 1640, fetal bovine serum (FBS) and penicillin/streptomycin solution, were obtained from Sigma-Aldrich (St. Louis, MO, USA); 0.05% trypsin ethylenediaminetetraacetic acid (EDTA) (1×) was obtained from Thermo Fisher Scientific.

### 2.2. Experimental Setup

The prepared samples were simultaneously measured using three analysis methods (the hemocytometer, commercial cell counter and NaviCell) and the results were compared. The staining steps and sample concentrations followed the protocols described below, which were recommended by each measurement method. The measurement results were then calculated considering the dilution factors depending on the staining of cells.

#### 2.2.1. Measurement Procedure Using the Hemocytometer

For the cell counting and viability testing using the hemocytometer, the cells were stained with a sample and trypan blue reagent mixing ratio of 1:1 for 1 min. First, the number of cells was counted in five 1 mm × 1 mm areas of the hemocytometer and the sum of the cells was divided by the number of measurement areas, i.e., five, to obtain the average number of cells. The total concentration was calculated as cells/mL by multiplying the average number of cells by 10,000 (volume and unit conversion factor) and the dilution ratio due to the dye. The 10 μL sample was injected onto the hemocytometer chip and analyzed manually with a microscope.

#### 2.2.2. Measurement Procedure Using the Commercial Cell Counter

To measure the samples using the commercial cell counter, the 10 μL sample was injected onto an exclusive commercial chip and the chip was then inserted into the instrument. The commercial cell counter has an auto focus function; hence, the samples can be analyzed automatically without further manipulation. The recommended staining protocol for preparing the samples is the same as that of the hemocytometer. The measurement results can be obtained without additional manual calculations because the instrument offers a dilution factor calculation option.

#### 2.2.3. Measurement Procedure Using the NaviCell

After injecting the 10 μL sample onto the dedicated chip, the chip was inserted into the NaviCell. Neither the focusing of optical devices nor staining of the samples was needed for the NaviCell; consequently, analysis results could be obtained within 13 s after chip insertion. In addition, the problem of cell image intensity dependence on dyeing time, which is manually controlled, is inherently prevented [24,29,30,31]. Furthermore, analysis results could be obtained within 13 s after chip insertion.

### 2.3. Preparation of Cell Lines for Experiment

#### 2.3.1. Cell Counting

To demonstrate the reagent-free cell counting performance of the NaviCell, we used 13 types of mammalian cell lines: (i) BT-474 (human breast invasive ductal carcinoma cell line derived from breast tissues); (ii) L-929 (mouse fibroblast cell line derived from connective tissue); (iii) MDA-MB-231 (human invasive ductal carcinoma cell line derived from breast tissues); (iv) SK-BR-3 (human invasive ductal carcinoma cell line derived from breast tissues); (v) SWRC-G-R-O (human renal carcinoma cell line derived from renal tissues); (vi) U-87 (human glioblastoma cell line derived from brain tissues); (vii) COS-7 (*Cercopithecus aethiops* fibroblast cell line derived from monkey kidney tissues); (viii) CHO (mouse epithelial cell line derived from the ovary of Chinese hamster); (ix) HELA (human cervical carcinoma cell line derived from cervix); (x) HL-60 (human promyelocytic leukemia cell line derived from peripheral blood); (xi) HT-29 (human adenocarcinoma cell line derived from colon epithelium); (xii) PC-3 (human prostate carcinoma cell line derived from prostate); and (xiii) A549 (human lung carcinoma cell line derived from lung tissue). SWRC-G-R-O was obtained from the Samsung Medical Center (Seoul, Korea) and the other cell lines were obtained from the American Type Culture Collection (ATCC; Manassas, VA, USA). Three types of cell lines (U-87, COS-7 and HELA) were grown in a DMEM, while the remainder were grown in a RPMI 1640. Then, 10% heat-inactivated FBS and 1% penicillin/streptomycin solution were added to both culture mediums to maintain a stable environment.

#### 2.3.2. Cell Viability

To analyze the reagent-free viability assay, we used six cell lines, i.e., BT-474, L-929, MDA-MB-231, SWRC-G-R-O, U87 and THP-1 (human acute monocytic leukemia cell line derived from monocyte). The THP-1 cell was acquired from the ATCC. The cells were maintained in RPMI 1640 containing 10% FBS, 1% penicillin/streptomycin solution under 95% relative humidity and 5% CO_2_ at 37 °C. All cell lines were transferred to ep-tubes and left at room temperature. These cell lines eventually died naturally over time. The number of dead cells was manually counted every 6 h. When the viability approached 50%, the cell line was used in the experiments. Depending on the cell types, the viability reduction times varied from 24 h to 48 h. The viability (%) was calculated with Equation (1).

(1) $$\mathrm{Viability}\;\left(\%\right)=\frac{\left(Live\;cell\;count-Dead\;cell\;count\right)}{Live\;cell\;count}\times 100\%,$$

#### 2.3.3. Cell Harvesting

Since the analysis methods used in this study require samples to be prepared on separate chips, all cell lines for experiments must be harvested prior to the experiments. Among the 13 cell lines, two cell lines, HL-60 and THP-1, can be easily transferred to the ep-tubes without harvesting because they are spontaneously suspended, while reagents for detaching should be added for harvesting for the other cells. For harvesting the adherent cells, 100 μL of 0.05% trypsin-EDTA (1×) was added to 12 well plates and the cells were then incubated in the CO_2_ incubator for 1 min at 37 °C. To deactivate the trypsin, 1 mL serum containing 10% FBS and 1% pen/strep was mixed in the well plate. The deactivated samples were gently re-suspended and transferred to the ep-tubes. The ep-tube containing each cell line was centrifuged for 5 min at 300× *g*. After discarding the supernatant of the samples, a fresh medium was added to prepare the sample with cell concentrations ranging from 1 × 10^6^ to 4 × 10^6^ cells/mL.

## 3. Results and Discussion

### 3.1. Analysis Algorithm

**Figure 2:**
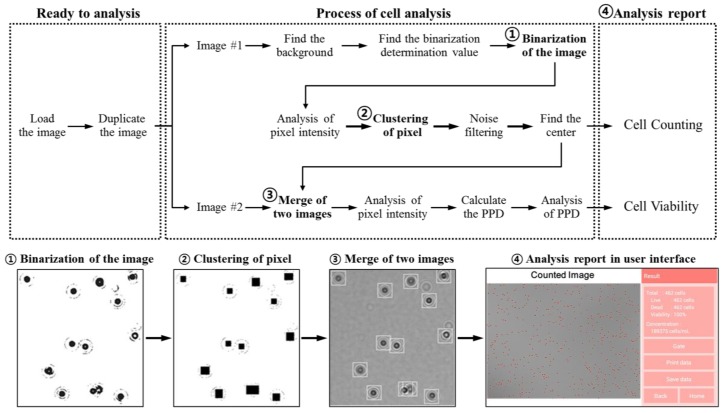
Concise workflow of the algorithm for the processing of the lens-free shadow image. The algorithm consists of binarized image, clustering, counting and viability analysis. Bottom pictures show how the original image is transformed at critical steps.

The reported cell analyzer (Figure 1b) based on the LSIT (Figure 1a) can automatically analyze samples captured using the CMOS image sensor. To achieve optimal performance for automatic analysis, we developed a custom analysis algorithm for the cell counting and viability testing (Figure 2). The analysis begins by duplicating the photographed image into two identical images. First, the analyzer divides the stored image into 16 areas and obtains the average intensity value of each area. It then derives a binarization determination value, which is the difference between the average value and gray value which was empirically set for each kind of cell. By comparing the intensity of each pixel in the image with the binarization determination value, each pixel intensity is modified to 96 if it is less than the determination value and to 255 if it is greater than the determination value. The determination value distinguishes the shadow image signals that can be recognized as cells by primarily removing debris or contaminants smaller than the cells contained in the sample. Through this process, the original image is changed to the binarized image.

The image is now analyzed sequentially from the top left pixel of the binarized image. If the pixel value is 96, a matrix of 3 × 3 pixels is set around the pixel. Inside the matrix, all pixel values of 96 change to 175. At this time, if any pixel other than the center point has a value of 96, an additional matrix of 3 × 3 pixels is set on the point and this process is repeated until there is no matrix to be expanded. If the size of the expanded matrix is more than 20 × 20 pixels, it is detected as noise and the pixel values inside the matrix are changed to 255. However, among the matrices of 20 × 20 pixels or more that are detected as noise, a matrix of 20 × 40 pixels or less in a rectangular shape is recognized as having two cells attached and two matrices of 20 × 20 pixels that overlap with each other are formed. When cells form clumps or islands, they are detected as noise and are not counted as cells. This is because if the cells continue to clump after the trypsin process, they are judged to be abnormal cells. If the matrix size is 20 × 20 pixels or less, the center point of the matrix is set to 60 and all other pixels to a value of 255. Here, 20 × 20 pixels is the empirical optimal range for analyzing shadow images of tested cells in this paper. After this process, the original image is transformed into a binary image in which the points having a value of 60 are distributed on the background having the value of 255. The number of these points reflects the number of cells distributed in the sample. By merging the modified binary image over the previous original image, it is confirmed that the determined pixel points are located at the centers of the shadows of the cells.

Next, the analyzer finds the maximum and minimum values of the pixel intensity in the square (11 × 11 pixels) centered on the cell. The difference between this maximum value and the minimum value is the peak- to-peak distance (PPD) value that enables viability analysis. The viability of the cells is determined by comparing the PPD value obtained for each cell on the cell center point with the empirically set cell viability constant, i.e., viability analysis. All these analyzed results are displayed on the user interface of the cell analyzer. The user interface is based on an Android platform through the user interface of the display; the NaviCell provides all the analyzed results, such as the original shadow image, image-processed shadow image, cell size distribution, cell concentration and total cell viability separated by dead cells and live cells.

### 3.2. Cell Counting

**Figure 3:**
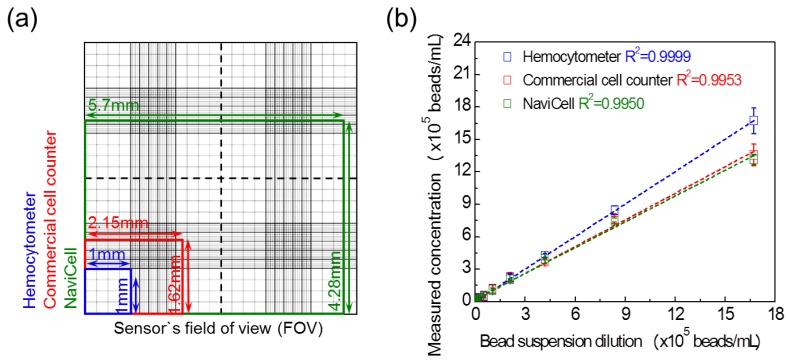
Advantages of the NaviCell platform. (**a**) FOVs of the three different measurement methods used in the experiment are superimposed for easy comparison. Blue box shows the FOV of a hemocytometer, red box shows the FOV of the commercial cell counter and green box shows the FOV of the NaviCell; (**b**) To measure 10 μm beads with various concentrations, the average standard deviation of the hemocytometer (blue line), the commercial cell counter (red line) and the NaviCell (green line) are 2.3 × 10^4^, 2.1 × 10^4^ and 1.1 × 10^4^ beads/mL, respectively.

The FOV represents the size of the area that can be measured at one time and is an important indicator for determining the performance of the cell counter. Figure 3a compares the FOV of the three measurement methods used in this study. The blue box shows the FOV of the hemocytometer (1000× microscope), which is 1 mm × 1 mm. The red box shows the FOV of the commercial cell counter with a size of 2.15 mm × 1.62 mm. The green box shows the FOV of the NaviCell with a size of 5.7 mm × 4.28 mm. The NaviCell therefore has an FOV approximately seven times and 24 times wider than the commercial cell counter and the hemocytometer, respectively. As expected, it is possible for the NaviCell to obtain the widest range of FOV because no optical lenses are installed.

Counting tests for 10 μm polystyrene beads using the three measurement methods demonstrated the counting performance of the NaviCell (Figure 3b). The bead concentration was measured as 1.6 × 10^6^ cells/mL using the hemocytometer, after which the samples with different concentrations were prepared by serial dilutions. As the measured R^2^ values of the trend lines for the three methods were 0.995 or more, it was first confirmed that all three measuring instruments were suitable counting instruments. The standard deviation (SD) values measured using the NaviCell were the smallest among the three measurement instruments. For example, when the bead concentration was 1.60 × 10^6^ cells/mL, the SD values of concentration were 1.19 × 10^5^ cells/mL, 1.01 × 10^5^ cells/mL and 0.48 × 10^5^ cells/mL measured by the hemocytometer, the commercial cell counter and the NaviCell, respectively. Since no optical lenses were installed in the proposed cell analyzer, it is possible to obtain a wider range of FOV than conventional cell counters based on microscopy. Based on the bead counting results, it is more likely that the wide FOV can contribute to the enhanced SD between the measurement results. The NaviCell has a detection range of 10^4^–10^6^ cells/mL (see Supporting Information (Figure S1)).

**Figure 4:**
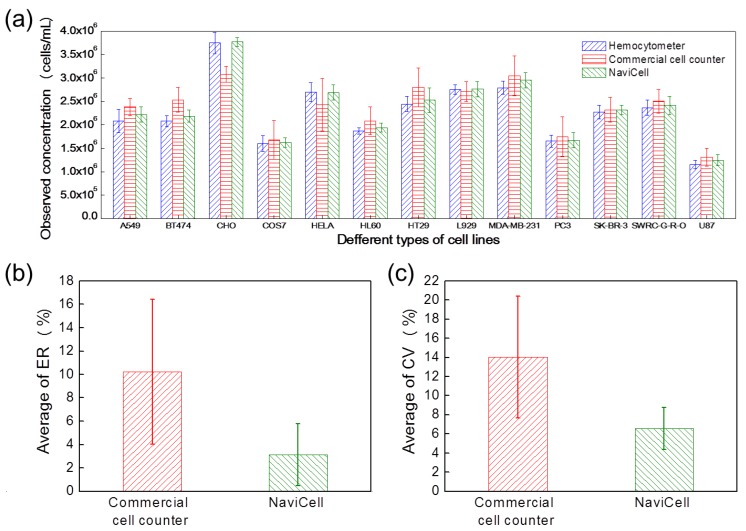
Comparison of cell counting performance using 13 cell lines. (**a**) Cell counting results of the NaviCell and the conventional methods are analyzed for the A549, BT474, CHO, COS7, HELA, HL60, HT29, L929, MDA-MB-231, PC3, SK-BR-3, SWRC-GRO and U87 cell lines. Improved counting precision of the NaviCell is demonstrated in terms of (**b**) average ER values and (**c**) average CV values.

Cell counting refers to the measurement of the number of cells in a certain volume and is the most essential function of the cell counter. Because cells vary in shape and size depending on the type and state of cells, tests for various types of cells are required to demonstrate the performance of cell counters. Figure 4a presents the analyzed results of 13 mammalian cell lines (A549, BT474, CHO, COS7, HELA, HL60, HT29, L929, MDA-MB-231, PC3, SK-BR-3, SWRC-GRO and U87) using the hemocytometer, the commercial cell counter and the NaviCell.

To compare the counting performance, the ER (Figure 4b) and CV (Figure 4c) were calculated for the measured 13 cell lines. The ER and CV were calculated using Equations (2) and (3), respectively.

(2) $$\mathrm{ER}=\frac{\left(\sum^{}x_{i}-\sum^{}x_{j}\right)}{\sum^{}x_{i}}\times 100\%,$$

where $x_{i}$ is the observed concentration counted by the hemocytometer and $x_{j}$ is the observed concentration counted by the commercial cell counter or the NaviCell.

(3) $$\mathrm{CV}=\frac{1}{µ}\sqrt{\frac{\Sigma_{m=1}^{n}{\left(x_{m}-µ\right)}^{2}}{n-1}}\times 100\%,$$

where $x_{m}$ are the observed concentrations of the sample items, *µ* is the mean value of these observations and n is number of observations in the sample. In this study, the cell measurements are repeated 10 times.

Here, the ER was calculated based on the results measured by the hemocytometer, which is the standard. The average ERs of the commercial cell counter and the NaviCell were 10.22 ± 6.21% and 3.13 ± 2.65%, respectively. The average CV values of the commercial cell counter and the NaviCell were 14.03 ± 6.37% and 6.58 ± 2.20%, respectively. This implies that the NaviCell can measure samples at a level closer to that of the standard compared to the commercial cell counter. Also, the NaviCell provides measured results with a lower SD, i.e., enhanced precision, than the conventional cell counter.

### 3.3. Cell Viability

Cell counting generally targets all of the cells in a sample without distinguishing between dead and live cells. However, the concentration of live cells in the total cell concentrations needs to be checked frequently to determine the cell viability. To perform the cell viability testing, the cells should be color-stained or fluorescently labeled. Since fluorescent labeling is a relatively expensive procedure, color staining has been widely used.

Trypan blue, one of the most widely used cell staining reagents, penetrates the cell membrane of damaged cells and then stains the cytoplasm blue. Therefore, the cells that are not stained are considered live, whereas the stained cells are considered dead. Although cell staining is inexpensive and easy to use, additional error is always a possibility when determining cell viability, depending on the pipetting and staining time.

**Figure 5:**
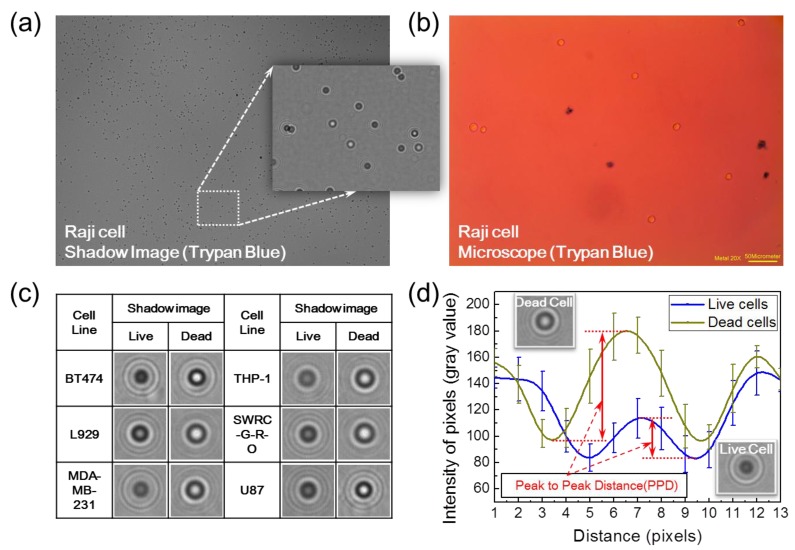
Principle of viability analysis without reagent. (**a**) Lens-free shadow image of stained Raji cells; (**b**) Corresponding microscope image of (**a**); (**c**) Live and dead cell shadow images of six cell lines (BT474, L929, MDA-MB-231, THP-1, SWRC-G-R-O and U87) without staining; (**d**) The PPD is defined as a difference between the highest and lowest intensity of all pixels in a specific square area, enabling the analysis of cell viability without reagent. Note that the analysis with the NaviCell does not require any staining procedure intrinsically; however, Figure 5a was inevitably acquired after the staining procedure in order to analyze the identical sample both with a microscope and the Navicell.

The suggested cell counter in this study enables the analysis of cell viability without pretreatment of cells. The NaviCell can analyze the contrast of the shadow image of a cell to distinguish the live and dead states. Figure 5a,b shows matching images taken with the NaviCell and the microscope. The Raji cell line was used for the experiment and was stained with trypan blue only for the conventional method. As can be seen in the matched images, the contrast of the shadow image of the dead cells (stained cells) increased up to 354% compared to living cells (unstained cells). To obtain the matched shadow image with the microscope (Figure 5a), this shadow image was also taken after staining.

To demonstrate the ability of the NaviCell to analyze the viability of non-stained cells, we obtained the shadow images of six different cell lines (BT474, L929, MDA-MB-231, THP-1, SWRC-GRO and U87). Figure 5c shows the representative shadow images of the live and dead cells, showing that the shadow image contrasts of the dead cells significantly differ from those of the live cells, even though none of the cells were stained.

The increase in the contrast of the shadow images is related to the changes in cell viability. A dead cell refers to a state in which a cell membrane is ruptured. Since the dead cell forms a relatively flat shape compared to a living cell, it is speculated that light from the LED source attenuates less as the light passes through the shorter path inside the dead cell, resulting in increased intensity of the shadow image. In order to realize the cell viability assay using the NaviCell, the appropriate reference of the contrast value for each cell line must be determined in advance, since the contrast of the shadow image changes depending on the type of cells. The contrast value was quantified as the peak-to-peak distance (PPD) (Figure 5d). The PPD is the difference between the highest and lowest intensity of all pixels, i.e., the zero-order peak and first-order valley in the *Airy* disk, in the square area (11 × 11 pixels). The reference values of PPD for the abovementioned six cell lines were studied using both microscope images and shadow images and were applied to the proposed cell analyzer.

**Figure 6:**
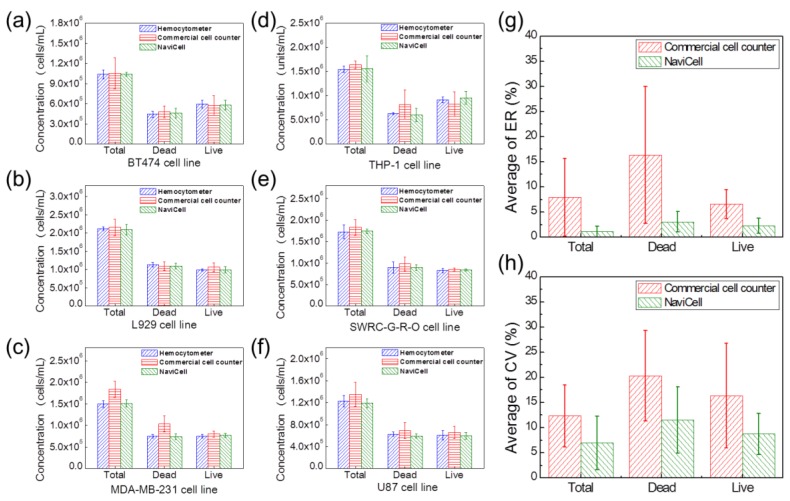
Comparison of cell viability performance using six cell lines. (**a**–**f**) Concentrations of BT474, L929, MDA-MB-231, THP-1, SWRC-G-R-O and U87 cell lines, respectively, by dividing into total number of cells, number of dead cells and number of live cells; (**g**) Average ER of the total cell count, dead cell count and live cell count; (**h**) Average CV of the total cell count, dead cell count and live cell count.

The cell viability performance of the NaviCell was evaluated using six cell lines, i.e., BT474 (Figure 6a), L929 (Figure 6b), MDA-MB-231 (Figure 6c), THP-1 (Figure 6d), SWRC-G-R-O (Figure 6e) and U87 (Figure 6f). The measurement results of the hemocytometer and the commercial cell counter were compared with those of the NaviCell. The cells were used in experiments when the cell viability approached 50% by manual count. Different cell concentrations and staining treatments were used following the recommended conditions for each measurement method, while the samples for the NaviCell were not stained at all. Concentration values were calculated by considering the dilution factor of the measured results depending on the number of dyes that were added.

To verify the viability testing results of the NaviCell, the ER and CV values defined above were derived for the total number of cells, number of live cells and number of dead cells, as shown in Figure 6g,h. The ER values were calculated based on the results measured by the hemocytometer, which is the standard.

In Figure 6g, the average ERs of the commercial cell counter are 7.95 ± 7.74% (total number of cells), 16.37 ± 13.63% (number of dead cells) and 6.55 ± 2.86% (number of live cells), while those of the NaviCell are 1.13 ± 1.09%, 3.07 ± 2.04% and 2.25 ± 1.52%, respectively. It has been confirmed that the Navicell results are three times closer to the standard than the commercial cell counter. In Figure 6h, the average CV values of the commercial cell counter are 12.31 ± 6.14% (total number of cells), 20.31 ± 8.98% (number of dead cells) and 16.34 ± 10.41% (number of live cells), while those of the NaviCell are 6.96 ± 5.33%, 11.52 ± 6.61% and 8.75 ± 4.08%, respectively. Therefore, the NaviCell has been proven to have one and half times better precision and repeatability than the commercial cell counter. More importantly, the ability to analyze cell viability without the use of a microscope and reagents was possible only by using the proposed cell analyzer.

## 4. Conclusions

In this paper, we developed a commercial-level field-portable lens-free cell analyzer (NaviCell) based on LSIT and demonstrated its performance. Various cell lines were analyzed for the cell counting and viability testing and compared to established measurement techniques, i.e., the hemocytometer and commercial cell counter. With 13 different cell lines, the ER and CV of the cells analyzed with the NaviCell were closer to the standard than those of the commercial cell counter. Furthermore, because the NaviCell has a larger FOV, the SD of the analysis data from the NaviCell was inherently lower than that of the hemocytometer and commercial cell counter. The cell viability performance of the NaviCell was also compared to typical analysis methods by using six different cell lines. The analysis of the ER and CV for the cell viability testing with the proposed cell counter was closer to the reference method than the commercial cell counter. Therefore, the NaviCell was evaluated to be a suitable alternative cell counter in terms of performance. Note that none of the measurements using the NaviCell required additional staining treatment. Because the NaviCell has no built-in optical parts of a conventional microscope, it has the following beneficial features: (1) reagent-free analysis; (2) enhanced SD owing to large FOV; (3) compact physical dimensions; (4) low cost; and (5) automated analysis for cell counting and viability testing. We therefore believe that the NaviCell can be considered as an advanced alternative tool in the fields of cell counting and viability testing in terms of both efficiency and precision.

## Figures and Tables

**Table 1:** Overview of the NaviCell and commercial cell counters.

	Gyrozen * (NaviCell)	Invitrogen (Countess I)	Invitrogen ** (Countess II)	Bio-Rad (TC-20)	Logos (Luna)	Logos (Luna II)	Nexcelom (Cellometer Auto T4)	Coulter’s Vi-CELL
Operating Power	100–240 V	100–240 V	100–240 V	90–240 V	110-240 V	100–240 V	100–240 V	100, 120, 220, 240 V
Cell Diameter (µm)	5 to 80	5 to 60	4 to 60 (count) 7 to 60 (viability)	6 to 50	3 to 60	3 to 60	5 to 60	5 to 70
Detection range (cells/mL)	10^4^–10^6^	10^4^–10^7^	10^4^–10^7^	5 × 10^4^–10^7^	5 × 10^4^–10^7^	5 × 10^4^–10^7^	10^5^–10^7^	5 × 10^4^–10^7^
Processing Time (second)	13	60	15	30	7	22 (autofocusing)	30	210
Dimensions (W × D × H) (mm)	162 × 135 × 138	270 × 200 × 190	228.6 × 139.7 × 228.6	190 × 150 × 254	220 × 210 × 90	160 × 180 × 280	89 × 107 × 320	380 × 410 × 445
Weight (Kg)	1	2.1	3.6	2.2	1.2	1.6	4.7	11.3
Camera	5 Mega Pixel	3.1 Mega Pixel (2.3× obj)	5 Mega Pixel (2.5× obj)	-	5 Mega Pixel	5 Mega Pixel	-	Manual-focus CCD array (1.4 Mega Pixel)
Field of View	5.70 mm × 4.28 mm (24.396 mm^2^)	2 mm × 2 mm (4 mm^2^)	2.15 mm × 1.62 mm (3.5 mm^2^)	2 mm × 2 mm (4 mm^2^)	-	-	-	-
Working principle	Lens-free	Microscopy	Microscopy	Microscopy	Microscopy	Microscopy	Microscopy	Microscopy
Reagent	Free	Required	Required	Required	Required	Required	Required	Required

*: Proposed lens-free cell analyzer in this paper; **: Commercial cell counter in this paper.

## Supplementary Materials

The followings are available online at http://www.mdpi.com/1424-8220/18/1/85/s1, Figure S1: Detected images of 10 μm beads by the NaviCell; original image of a concentration of 15,000 cells/mL (**a**), 120,000 cells/mL (**b**), and 1,300,000 cells/mL (**c**) acquired with the CMOS image sensor. (**d**–**f**) are the results of the analysis from each original image.
