# Supplementary material for: A Field-Portable Cell Analyzer without a Microscope and Reagents
Source: Sensors (Basel). 2017 Dec 29;18(1):85. doi: 10.3390/s18010085 (PMC5795886; doi:10.3390/s18010085)
Supplement: Supplementary file 1 [file sensors-18-00085-s001.pdf]

Supplementary Materials

Article

A field-portable cell analyzer without a microscope and reagents

Dongmin Seo [1,†], Sangwoo Oh [1,2,†], Moonjin Lee [2], Yongha Hwang [3,*], and Sungkyu Seo [1,*]

1 Department of Electronics and Information Engineering, Korea University, Sejong, Republic of Korea
2 Maritime Safety Research Division, Korea Research Institute of Ships & Ocean Engineering, Daejeon, Republic of Korea
3 Department of Electro-Mechanical Systems Engineering, Korea University, Sejong, Republic of Korea
† These authors equally contributed to this study.
* Correspondence: (S. Seo) E-mail; sseo@korea.ac.kr, Tel; +82-44-860-1427, (Y. Hwang) E-mail; hwangyongha@korea.ac.kr, Tel; +82-44-860-1442

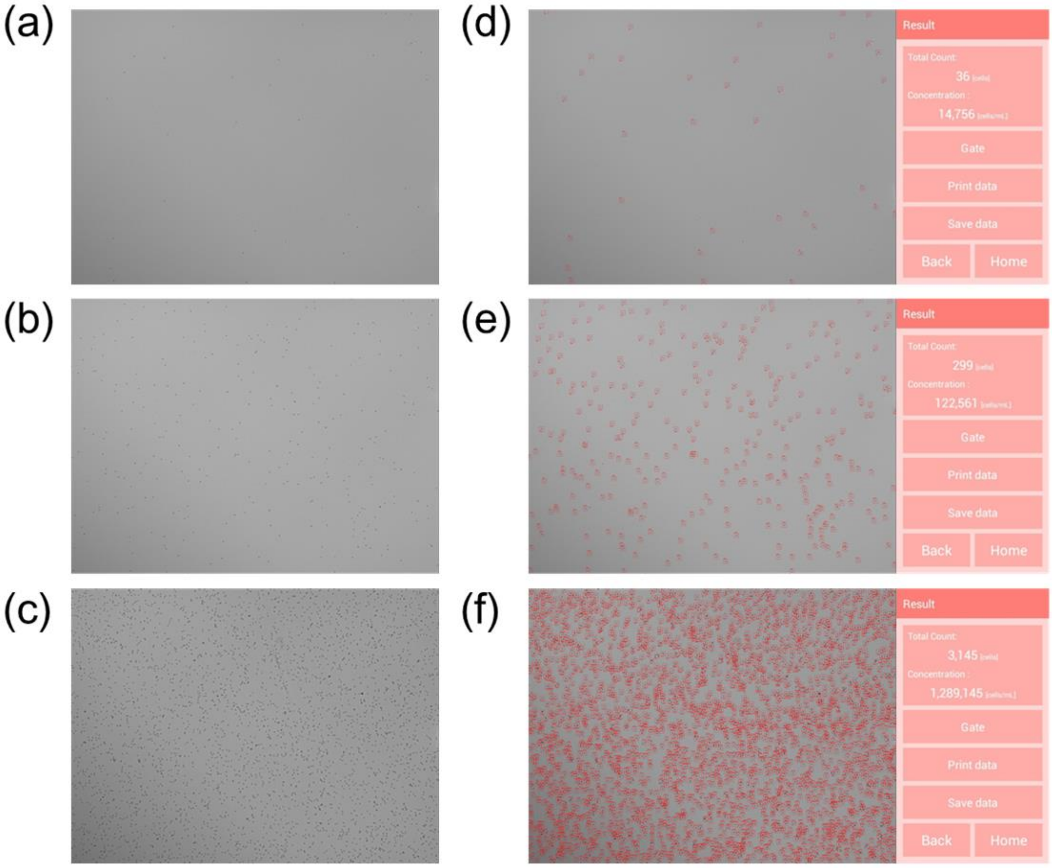

Figure S1. Detected images of 10 μm beads by the NaviCell; original image of a concentration of 15,000 cells/mL (a), 120,000 cells/mL (b), and 1,300,000 cells/mL (c) acquired with the CMOS image sensor. (d), (e), and (f) are the results of the analysis from each original image.
